# A Case Report of an Intestinal Helminth Infection of Human Hymenolepiasis in Rural Gambia

**DOI:** 10.23937/2378-3656/1410251

**Published:** 2019-01-04

**Authors:** Usman N Ikumapayi, Chilel Sanyang, Dora IA Pereira

**Affiliations:** 1 Medical Research Council Unit the Gambia, London School of Hygiene & Tropical Medicine, Banjul, The Gambia; 2 Department of Pathology, University of Cambridge, UK

**Keywords:** Tapeworm, Hymenolepiasis, *Hymenolepis nana*, The Gambia, Children

## Abstract

**Background:**

*Hymenolepis nana,* also called dwarf tapeworm infection, is an intestinal helminth not previously reported in The Gambia and only very rarely reported in West Africa.

**Case presentation:**

We report a case of *H. nana* infection in a 29-month-old child living in a rural community of the north bank of the Upper River Region (URR) in The Gambia. The child presented with mild iron deficiency anaemia and granulocytosis but was otherwise mostly asymptomatic despite the moderate-intensity of infection.

**Conclusions:**

We support treatment of *H. nana* infection even in largely asymptomatic children to prevent autoinfection and spread of this intestinal helminth in The Gambia and in other West African countries.

**Abbreviations:**

GCP: Good Clinical Practice; HAZ: Height-for-age z-score; IHAT-GUT: Acronym for the Iron Hydroxide Adipate Tartrate Supplementation Study; ICH: International Conference on Harmonisation; SD: Standard Deviation; URR: Upper River Region; WAZ: Weight-for-age z-score; WHO: World Health Organization; WHZ: Weight-for-height z-score

## Background

Hymenolepiasis is a neglected tropical disease and a type of helminthiasis infection which in humans is most commonly caused by *Hymenolepis nana,* often referred to as dwarf tapeworm. Hymenolepiasis frequently occurs in arid, warm and resource-poor regions [1-3], with the highest prevalence observed in children under 15-years-old, and an estimated 50 - 75 million carriers worldwide [4-7]. Very few reports of *H. nana* infection exist in the literature. There is a report of *H. nana* present in 28.6% of children living in the rural village of Touguri in Burkina Faso in 2011 [8]. Another report, in Ethiopia, showed that *H. nana* infection was present in 7.4% of children and that children infected with intestinal helminths had low haematocrit [9]. However, human infection with *H. nana or H. diminuta* have not been reported before in The Gambia or indeed in most West African countries and appears much more common in South East Asia [10].

*H. nana* belongs to the family Hymenolepididae and differs from all other human tapeworms because it does not require an intermediate host since the parasite is able to complete its entire cycle in a single host [11]. Due to this trait, the majority of infections occur as auto-infections, particularly in immune- compromised hosts, as a result of ingesting water or food contaminated by human faeces containing eggs [12]. Eggs passed in stool are immediately infective and this human-to-human transmission of eggs by the faecal-oral route is the most common route of infection, particularly in environments with poor hygiene and inappropriate sanitation. The ingested eggs hatch in the small intestine releasing a motile embryo, the oncosphere, which invades a villus and develops into the larval cysticercoid in about 4 weeks. The cysticercoid then ruptures the villus and attaches to the mucosal surface to mature into the adult tapeworm, the whole process takes approximately four weeks [11]. This parasite also has an indirect life cycle with humans and rodents as definitive hosts and arthropods, such as beetles and fleas, serving as intermediate hosts or vectors [10]. Accidental ingestion of contaminated food with infected arthropods is another possible route of infection. However, data on cases of hymenolepiasis attributed to food-borne infection are limited.

Mild infection with *Hymenolepis nana* is usually asymptomatic whilst heavy infection (> 500 eggs/g of stool), can cause severe morbidity in children, including anaemia, abdominal pain, diarrhoea, nausea, vomiting, headache, chronic urticaria, skin eruption, flatulence, weight loss, irritable behaviour and reduced growth [13-16]. The diagnosis of *H. nana* is usually made via microscopic identification of eggs in stool specimens [16].

Here we describe the first report in the literature of *H.nana* infection in a young Gambian child. To our knowledge, there are no other reports of hymenolepiasis in The Gambia.

## Case Presentation

**Figure 1:**
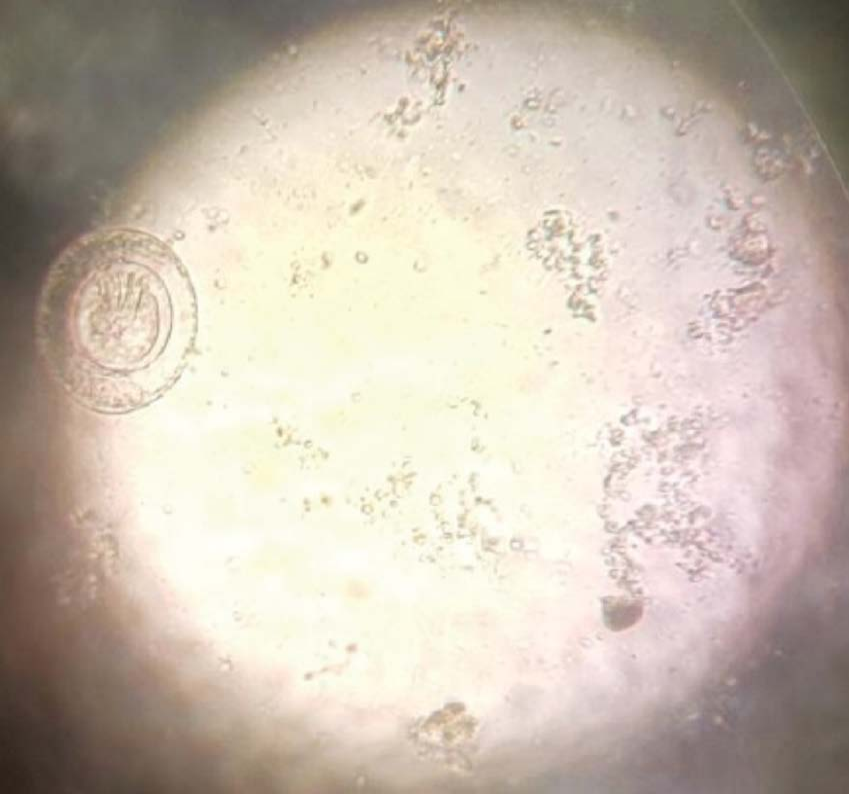
Image of the parasite identified as *Hymenolepis nana.* This picture was taken using a mobile phone facing the eyepiece whilst the object remains focused on the microscope stage with x 40 objective. Eggs were 30-50 pm and egg morphology was confirmed as representative of *H.nana* eggs based on CDC guidelines (https://www.cdc.gov/dpdx/hymenolepiasis/index.html).

In our routine screening of 340 children, aged 6-35 months old and enrolled in an iron supplementation trial (namely IHAT-GUT, ClinicalTrials.gov identifier: NCT02941081), we found the case of a 29-month-old child with moderate infection of *Hymenolepis nana.* As part of the trial protocol, we examined 521 stool samples from study participants for intestinal parasites, using the Kato-Katz method based on duplicate slides as per current WHO recommendation [17]. The child with the stool sample harbouring *H. nana* eggs (Figure 1) was unique amongst the 72 children out of 340 (21%) in whom intestinal parasites were present. The child presented with an infection intensity of 144 eggs/gram of stool and was mildly anaemic, with an Hb value of 10.8 g/dl, and iron deficient, with ferritin of 12.9 µg/l. The child also had a high white blood cell count and high granulocyte count, indicative of infection (Table 1). Otherwise, the child was growing normally (Table 1) and, upon examination by the study nurses, was considered as an asymptomatic carrier of hymenolepiasis.

**Table 1:** Demographic characteristics of the child infected with *Hymenolepis nana*.

Characteristic	Result*	Normal range
Age (months)	29	
WAZ (weight-for-age)	-1 SD	- 2 SD - + 2SD
HAZ (height-for-age)	0 SD	- 2 SD - + 2SD
WHZ (weight-for-height)	-1 SD	- 2 SD - + 2SD
Haemoglobin (g/dl)	**10.8**	11.5 - 16.5
Mean Cell Volume (fl)	**72.7**	75.0 - 100.0
Haematocrit (%)	**32.2**	35.0 - 55.0
Red Blood Cells (x 10^−12^/L)	4.42	3.50 - 5.50
White Blood Cells (x 10^−9^/L)	**17.8**	3.5 - 10.0
Granulocytes (x 10^−9^/L)	**12.5**	1.2 - 8.0
Ferritin (pg/l)	**12.9**	> 30

* Result: Outside the normal range for children < 5 years in The Gambia are in bold.

To prevent the spread of the infection, and irrespective of the asymptomatic status, the infected child was treated with praziquantel 20 mg/kg in a single oral dose following WHO guidelines, and was re-assessed 2 weeks later and considered free of the cestode.

## Discussion and Conclusions

*Hymenolepis nana* is a rare intestinal cestode in The Gambia despite being one of the most common intestinal helminths worldwide, particularly in poor communities in warm and arid countries [11,18]. The dwarf tapeworm is among the few intestinal worms that can have a reproductive life cycle without requiring an intermediate host [19]. This ontogenetic aspect of the worm can lead to hyper-infection, with autoinfection persisting for many years if left untreated [14,20].

Human *H. nana* infection is presumed to be mostly asymptomatic for intensities of up to 500 eggs/g of stool [13,14]. Indeed, in our investigation of this case, we confirmed that the child was largely asymptomatic, despite the moderate worm burden, and did not present with diarrhoea or abdominal pain or any other gastrointestinal symptom that has been associated with *H. nana* infection in children [16,18,21]. However, the child presented with anaemia and high white blood cell count due to high numbers of granulocytes, possibly eosinophilia, both conditions previously reported in indigenous Australian indigenous young children as as sociated with *H. nana* infection [16]. Furthermore, it is possible that even this moderate-intensity of *H. nana* infection has contributed to the iron deficiency anaemia diagnosis in this child, possibly due to impaired iron absorption secondary to the inflammation caused by the parasitic infection. Additionally, during the follow-up visit to provide the child with praziquantel treatment, the nurses conducted a thorough interview with the mother and found that the child usually showed irritable behaviour and difficulty in settling down, as well as occasional diarrhoea, all symptoms previously associated with high-intensity of *H. nana* infection [13].

Rural Gambia is not known to be an endemic nor a pandemic region for hymenolepiasis. Some studies have associated environmental conditions with the spread of *H. nana,* particularly warm climate and temperate zones and remoteness [2,3,22]. The north bank of the URR in The Gambia is a rural and remote region reaching extreme high temperatures (36 °C - 44 °C) from February to June each year, and it is possible that these factors may have favoured the transmission and persistence of the parasite in the community. We speculate that this child was infected initially via contact with contaminated food or faeces, maybe from a foreigner travelling in the area, and that due to autoinfection, the infection intensity increased over time. Praziquantel, the recommended treatment for hymenolepiasis, is not routinely administered in The Gambia as part of the national deworming programme with mebendazole, which is largely ineffective against tapeworms [23]. This probably explains why the autoinfection was allowed to continue even when pre-school age children receive deworming tablets every 6 months.

Since the national deworming programmes do not target *H. nana,* we decided to treat all members of the household with praziquantel, following WHO treatment guidelines, as a measure to control the spread of this rare infection in that community.

We report the first case of *H. nana* infection in The Gambia where this worm is thought to be very rare. Due to the likelihood of autoinfection this tapeworm can reach very high infection intensity over time if left undiagnosed and untreated. Our investigation has shown possible spread of *H. nana* infection within remote rural communities in the absence of treatment. Hence, it is important to screen *H. nana* alongside other intestinal parasites and to treat even if infection is thought to be asymptomatic.

## Data Availability

All data generated or analysed during this study are included in this published article.
